# Esophageal Perforation Following C4–C6 Anterior Cervical Corpectomy With Anterior Plate Fixation: A Rare but Life‐Threatening Complication Requiring Surgical Repair

**DOI:** 10.1002/ccr3.71813

**Published:** 2026-01-02

**Authors:** Maisam Gharayba, Nuha Riyad, Basem Bali, Mohammed Maree, Karam Rabi, Zaid Sawaftah, Osama Ewidat

**Affiliations:** ^1^ Department of Medicine An Najah National University Nablus Palestine; ^2^ Makassed Charitable Society Hospital East Jerusalem Palestine; ^3^ Department of General Surgey Ibn Sina Specialized Hospital Jenin Palestine

**Keywords:** anterior cervical corpectomy and fusion, anterior cervical plate fixation, cervical spine trauma, delayed postoperative complication, esophageal perforation, postoperative complications, spinal fusion

## Abstract

Anterior cervical corpectomy and fusion (ACCF) with anterior plate fixation is a common procedure for cervical spine trauma. Although generally safe, it carries a risk of esophageal perforation—a rare but potentially fatal complication. We report an 18‐year‐old male who developed delayed esophageal perforation 5 weeks after C4–C6 ACCF. Diagnosis was established by computed tomography showing retropharyngeal air and endoscopy confirming a full‐thickness esophageal defect. Surgical removal of the hardware, primary repair with absorbable sutures, drainage, and feeding jejunostomy were performed. The patient achieved complete recovery with no leakage at six‐month follow‐up. This case underscores the importance of clinical vigilance and timely surgical management.

## Key Clinical Message

This case highlights delayed esophageal perforation 5 weeks after anterior cervical corpectomy with anterior plate fixation in a young patient. Early recognition, prompt surgical intervention, and vigilant long‐term follow‐up are crucial to prevent life‐threatening complications.

## 1 Introduction

Anterior approaches to the cervical spine, first introduced in the 1950s, remain effective for managing trauma and degenerative conditions [1]. However, rare complications such as esophageal perforation may result in severe morbidity or mortality. The estimated incidence of esophageal injury during anterior cervical surgery is 0.2%–0.5%, and reported mortality ranges from 6% to 34% depending on the timing of diagnosis and management [2]. Early recognition and prompt intervention markedly improve survival and long‐term outcomes [3].

Anterior cervical procedures such as anterior cervical discectomy and fusion (ACDF), cervical disc arthroplasty (CDA), and corpectomy are considered safe, with low complication rates in large series [4].

Nevertheless, delayed esophageal perforation remains one of the most devastating complications, often caused by mechanical irritation from plates or screws.

Here, we report a unique case of delayed perforation in an 18‐year‐old male 5 weeks after C4–C6 anterior cervical corpectomy and fusion with anterior plate fixation, emphasizing diagnostic challenges, management strategies, and preventive considerations [4].

## 2 Case History, Examination and Management

An 18‐year‐old male with no significant past medical history sustained a C5 vertebral fracture after a diving accident, leading to complete spinal cord transection and quadriplegia.

He underwent anterior cervical corpectomy and fusion (ACCF) with anterior plate fixation spanning C4–C6. Postoperatively, he required a tracheostomy for airway management and a percutaneous endoscopic gastrostomy (PEG) tube for enteral nutrition.

**FIGURE 1:**
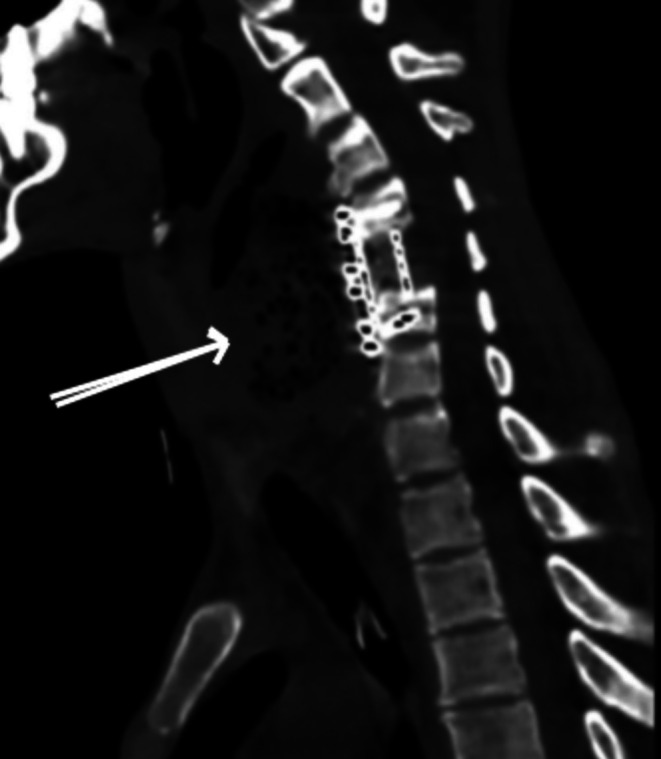
Cervical computed tomography demonstrating retropharyngeal air adjacent to the anterior cervical plate, suggestive of esophageal perforation.

Five weeks post‐surgery, the patient developed new‐onset neck pain, dysphagia, and localized swelling. He remained afebrile and hemodynamically stable, and there were no initial signs of systemic infection. These atypical and subtle symptoms in a tracheostomized, PEG‐fed quadriplegic patient raised suspicion for a delayed postoperative complication, most notably esophageal perforation. A non‐contrast cervical computed tomography (CT) demonstrated abnormal retropharyngeal air adjacent to the anterior cervical plate, raising concern for esophageal injury (Figure 1).

Subsequent upper gastrointestinal endoscopy confirmed a 1 × 1 cm full‐thickness perforation at the level of the upper esophageal sphincter, accompanied by a purulent fistulous tract. The esophageal lumen appeared divided into a true lumen and a fistulous tract.

Surgical exploration was promptly performed.

**FIGURE 2:**
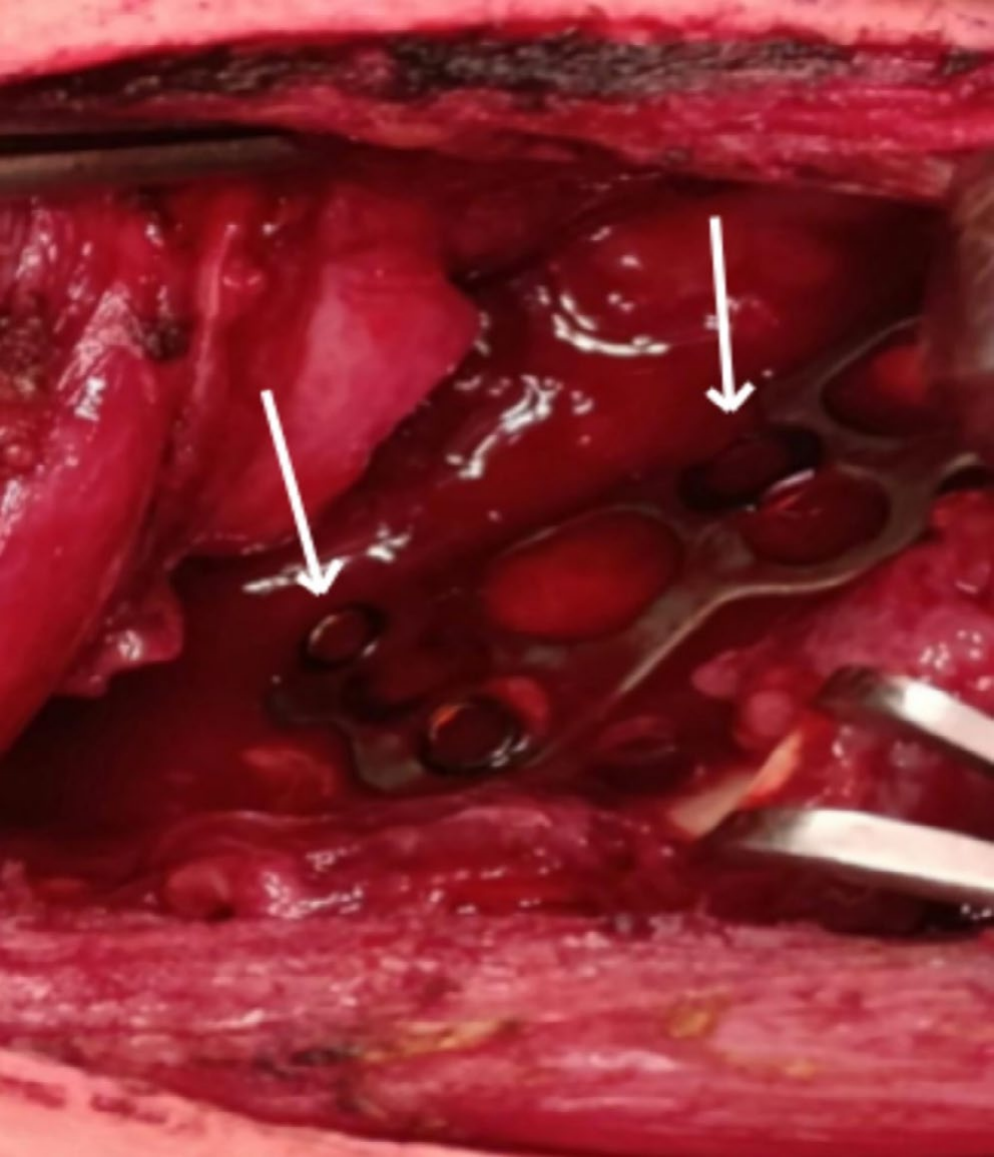
Intraoperative photograph showing erosion of the anterior cervical plate through the posterior wall of the esophagus.

The anterior cervical hardware was found to have eroded through the posterior wall of the esophagus (Figure 2). The plate and screws were carefully removed, and the defect was repaired in multiple layers using interrupted 3–0 Vicryl absorbable sutures.

A surgical drain was placed adjacent to the repair site, and a feeding jejunostomy tube was inserted to maintain nutrition during healing.

Broad‐spectrum intravenous antibiotics—vancomycin, meropenem, and ciprofloxacin—were initiated to cover oropharyngeal and enteric flora pending culture results.

Wound cultures revealed mixed aerobic flora sensitive to meropenem.

Antibiotic therapy was continued intravenously for 21 days, followed by 7 days of oral levofloxacin. Serial inflammatory markers demonstrated steady improvement, with C‐reactive protein decreasing from 126 mg/L preoperatively to 4 mg/L at discharge.

A postoperative barium swallow study at 3 weeks showed no contrast leakage, confirming the integrity of the esophageal repair.

The patient was maintained nil per os (NPO) until follow‐up imaging confirmed complete healing.

Oral intake was gradually reintroduced and tolerated well. At six‐month follow‐up, the patient remained clinically stable and free of dysphagia, infection, or hardware‐related complications.

Radiographs demonstrated stable cervical alignment without pseudoarthrosis.

Neurologically, he remained quadriplegic due to the initial spinal cord injury but had achieved improved sitting balance and partial self‐care capability through rehabilitation.

## 3 Differential Diagnosis, Investigations, and Treatment

The differential diagnosis included postoperative infection, retropharyngeal abscess, hardware displacement, and esophageal perforation.

A non‐contrast cervical computed tomography scan demonstrated abnormal retropharyngeal air adjacent to the anterior cervical plate, raising suspicion for esophageal injury (Figure 1). Upper gastrointestinal endoscopy subsequently revealed a 1 × 1 cm full‐thickness esophageal perforation at the level of the upper esophageal sphincter with a purulent fistulous tract.

Urgent surgical exploration was performed. Intraoperatively, the anterior cervical plate and screws were found to have eroded through the posterior esophageal wall (Figure 2). The hardware was removed, and the esophageal defect was repaired using multilayer interrupted 3–0 Vicryl absorbable sutures. A surgical drain was placed adjacent to the repair site, and a feeding jejunostomy tube was inserted.

Broad‐spectrum intravenous antibiotics (vancomycin, meropenem, and ciprofloxacin) were initiated. Wound cultures grew mixed aerobic flora sensitive to meropenem. Antibiotic therapy was continued intravenously for 21 days, followed by oral levofloxacin for 7 days.

## 4 Results, Outcome, and Follow‐Up

Serial inflammatory markers demonstrated marked improvement, with C‐reactive protein decreasing from 126 mg/L preoperatively to 4 mg/L at discharge.

A contrast barium swallow study performed 3 weeks postoperatively showed no evidence of contrast leakage, confirming successful esophageal healing. The patient was maintained nil per os until imaging confirmed integrity of the repair, after which oral intake was gradually reintroduced and well tolerated.

At six‐month follow‐up, the patient remained clinically stable with no dysphagia, infection, or recurrence of esophageal injury. Cervical spine radiographs demonstrated maintained alignment and stable fusion without pseudoarthrosis. Neurologically, the patient remained quadriplegic due to the initial injury but showed functional improvement through rehabilitation.

## 5 Dscussion

Delayed esophageal perforation following anterior cervical corpectomy and fusion (ACCF) is an uncommon but life‐threatening complication.

The diagnosis is often delayed because clinical symptoms are subtle and nonspecific, and the esophagus lies deeply within the prevertebral space, shielded by soft tissues.

Patients may initially present with dysphagia, odynophagia, neck pain, subcutaneous emphysema, or recurrent aspiration, which should immediately raise suspicion.

Intraoperative perforations typically result from direct mechanical trauma by retractors, surgical instruments, or nasogastric tubes [1, 3]. However, delayed injuries, as in our case, are most often caused by chronic pressure and friction from the anterior cervical plate or screws eroding into the posterior wall of the esophagus [5].

In this patient, continuous mechanical irritation from the cervical plate was the likely mechanism of gradual tissue necrosis and perforation.

Early and accurate diagnosis is crucial.

Computed tomography is a valuable first‐line imaging tool that can detect retropharyngeal air or fluid collections, while endoscopy directly visualizes the mucosal defect and confirms the diagnosis [6].

Our findings were consistent with this, as CT revealed retropharyngeal gas, and endoscopy identified a 1 × 1 cm full‐thickness perforation.

The management of delayed esophageal perforation after cervical surgery depends on the timing, size, and extent of injury.

Surgical repair remains the preferred approach for symptomatic or large perforations (> 1 cm), while conservative therapy may be reserved for very small, contained injuries [7].

In our patient, prompt surgical exploration, plate removal, multilayer primary closure, and drainage resulted in full recovery.

This aligns with the open management techniques described by [8], which emphasize debridement, drainage, and hardware removal as essential components of successful treatment.

Antibiotic therapy should be broad‐spectrum to cover oropharyngeal and enteric flora, as polymicrobial infections are common.

In our case, triple coverage (vancomycin, meropenem, ciprofloxacin) was justified, and infection control was confirmed by normalization of C‐reactive protein levels.

Fusion stability is another concern following hardware removal [9, 10].

Because the patient underwent plate removal only 5 weeks after corpectomy, posterior fixation was considered but deferred to prevent further soft‐tissue trauma.

Follow‐up imaging confirmed solid fusion and maintained spinal stability without pseudoarthrosis [4, 6].

Preventive strategies are critical to minimizing such complications.

These include ensuring that plates are positioned flush with the vertebral surface, minimizing screw protrusion, interposing soft tissue between the esophagus and the plate, and maintaining long‐term postoperative surveillance for dysphagia or neck swelling [6].

Finally, the absence of intraoperative or endoscopic photographs represents a limitation of our report.

Nonetheless, the case provides valuable insight into diagnostic and therapeutic decision‐making, especially in resource‐limited environments.

At six‐month follow‐up, the patient remained clinically stable, with complete esophageal healing and no signs of recurrence or infection.

## 6 Conclusions and Results

Esophageal perforation following anterior cervical corpectomy and fusion (ACCF) is a rare but potentially life‐threatening complication. Early recognition and timely surgical management are critical to preventing severe adverse outcomes, including sepsis and mortality. In this case, an 18‐year‐old male developed esophageal perforation after ACCF performed for a traumatic C5 cervical fracture resulting in quadriplegia. The emergence of neck pain, swelling, and dysphagia raised clinical suspicion and prompted thorough diagnostic evaluation. This case underscores the necessity of vigilant postoperative surveillance and highlights the imperative for rapid diagnosis and intervention to effectively manage complications and optimize patient outcomes.

## Author Contributions

**Maisam Gharayba:** conceptualization, data curation, resources, software. **Nuha Riyad:** investigation, project administration, supervision. **Basem Bali:** investigation, methodology, visualization, writing – original draft. **Mohammed Maree:** methodology, project administration, supervision. **Karam Rabi:** methodology, project administration, resources, software. **Zaid Sawaftah:** data curation, visualization. **Osama Ewidat:** methodology, project administration, writing – original draft, writing – review and editing.

## Funding

The authors have nothing to report.

## Ethics Statement

Our institution does not require ethical approval for reporting individual cases. This study was performed in accordance with the Helsinki Declaration of 1964 and its later amendments.

## Consent

Written informed consent was obtained from the patient for their anonymized information to publish this report in accordance with the journal's patient consent policy.

## Conflicts of Interest

The authors declare no conflicts of interest.

## Data Availability

All relevant data supporting the findings of this case report are included within the article. Additional details are available from the corresponding author upon reasonable request.
